# Solitary fibrous tumors of the soft tissues: imaging features with histopathologic correlations

**DOI:** 10.1186/2045-3329-3-1

**Published:** 2013-01-25

**Authors:** Zafaria G Papathanassiou, Marco Alberghini, Piero Picci, Eric Staals, Marco Gambarotti, Francesco Giuseppe Garaci, Daniel Vanel

**Affiliations:** 1 The Rizzoli Institute, via del Barbiano 1/10, 40136, Bologna, Italy

**Keywords:** Solitary fibrous tumor, Magnetic resonance imaging, Soft tissue tumor, Computed tomography, Soft tissue tumor

## Abstract

**Purpose:**

To describe the imaging features of soft tissue solitary fibrous tumors, with histopathological correlations and clinical outcome.

**Material and methods:**

Twenty-seven patients with histologically proven SFTs were retrospectively evaluated. Imaging studies included six radiographs, five U/S studies, eighteen CT scans, fourteen MRI exams, and one angiography.

**Results:**

On CT scans, two lesions were isodense and five were mildly hypodense compared to muscle while 11 lesions appeared heterogeneous-mixed of iso and hypodense areas. Heterogeneous enhancement was depicted in 13 lesions and four lesions enhanced homogeneously. Six lesions were partially calcified. On T1W MR images, seven lesions were isointense and one was slightly hyperintense relative to adjacent muscles while five lesions appeared heterogeneous-mixed of iso and hypointense areas. T2W images showed high SI in two cases and heterogeneous-mixed in seven cases. Enhancement was heterogeneous in six and homogeneous in four lesions. Patchy unenhanced areas (on CT and T1W MR images) along with patchy areas of low to markedly high SI on T2W images were depicted in 19 lesions. The enhanced portions correlated to areas of increased vascularity and cellularity. The four clinically more aggressive lesions could not be predicted on imaging.

**Conclusion:**

Typical soft tissue SFTs are deep masses made of isodense and isointense areas relative to adjacent muscles mixed with hypodense and hypointense areas on unenhanced CT and MR T1W respectively. Variable enhancement patterns and mixed to high signal intensities on MRT2W are attributed to tumor’s cellularity, vascularity, collagen distribution and/or degeneration. Heterogeneity of SFTs affects imaging features on MRI and CT modalities. The biological behavior of soft tissue SFTs can not be predicted based solely either on histopathologic or imaging evaluation.

## Introduction

Solitary fibrous tumors (SFTs) are spindle cell neoplasms that most commonly occur in the pleura and represent less than 5% of all pleural tumors [1]. In 1931, Klemperer and Rabin were the first to describe them as localized fibrous mesotheliomas [2]. Their histological origin has been controversial [3,4]. Pleural SFT is a well-established and generally benign entity with 7% -13% of cases being malignant at histopathological examination [5-7].

Even though SFTs are predominantly regarded as rare pleural neoplasms, a wide spectrum of extrathoracic sites has been reported, such as the head and neck regions [8-10], the abdomen, retroperitoneum and pelvis [11-17] and the soft tissues of the extremities [18-33]. Extrathoracic SFTs are rare and account for approximately 0, 6% of all soft tissue tumors [30-32]. Like their pleural counterparts, extrathoracic SFTs usually behave in a benign fashion but in approximately 10% of cases recur locally or metastasize [22,24]. So, an unusual tumor at an unusual location can be often poorly recognized affecting adversely the patient’s prognosis. Only few related studies predominantly with clinical and histopathological features have been reported (Table 1). Herein, we present, to the best of our knowledge, the largest retrospective imaging review of 27 histologically-proven cases of SFTs in the soft tissues.

**Table 1:** Review of the literature

	***SERIES***	***No of cases of soft tissue SFTS***
1	O’Connel JX et al. (1995) [18]	1
2	Suster S et al. (1995) [19]	2
3	Nielsen GP et al. (1997) [20]	4
4	Mentzel T et al. (1997) [21]	5
5	Vallat-Decouvelaere AV et al. (1998) [22]	2
6	Abe S et al. (1999) [23]	1
7	Hasegawa T et al. (1999) [24]	4
8	Harrigton P et al. (1999) [25]	1
9	Krismann M et al. (2000) [26]	1
10	Riss O et al. (2000) [27]	1
11	Akisue T et al. (2003) [28]	4
12	Rakheja D et al. (2004) [29]	1
13	Anders JO et al. (2006) [30]	1
14	Daigeler A et al. (2006) [31]	4
15	Martorell M et al. (2007) [32]	1
16	Cranshaw IM et al. (2009) [33]	13

## Materials and methods

From 1997 to 2009, twenty seven patients were diagnosed with soft tissue SFTs in our institution. Intra-abdominal or retroperitoneal SFTs were not included in this study because Rizzoli Institute is a specialized orthopedic center. Data concerning age, sex, physical examination, lesion number, size and site was recorded. Assessment of the available pre-operative imaging examinations was performed and included plain radiography (X-ray), ultrasound (U/S), digital substracted angiography (DSA), computed tomography (CT) and magnetic resonance imaging (MRI). Two patients were examined with X-rays, CT scans and MR imaging and four underwent only CT scans and MR imaging. Three patients had X-rays and CT scans and six had only CT scans. One case was investigated with X-rays and MR imaging and five had only MR imaging. Two patients underwent U/S and CT scans while two had U/S and MR imaging and another one had only U/S. Only one patient underwent CT scan and DSA. All CT scans were performed before and after contrast medium intravenous administration, except for one case that didn’t have post contrast images. Dynamic contrast medium infusion was carried out in one case and enhanced CT scans were obtained at 30 s, 60 s, 90 s and 180 s after injection. MRI examinations obtained from referring institutions as well included a variety of T1 weighted spin-echo (T1WSE), T2 weighted spin-echo (T2WSE), T2 weighted fast spin-echo with fat suppression (T2 FSE Fat Sat), short T1 inversion recovery (STIR) and T1WSE with fat suppression sequences (T1 SE Fat Sat). T1-weighted and Post gadolinium images were not acquired in one and four cases respectively. Radiological evaluation included lesion size, location, and internal morphology along with CT density, MR signal intensity and homogeneity, which were compared to neighboring muscles. Additionally, enhancement patterns, U/S echogeneity and radio-opacity were recorded. All of the lesions were surgically treated except for the most recent one. Histopathological analysis was based on macroscopic evaluation of the resected tumors, hematoxylin and eosin staining and immunohistochemistry (CD34, CD99, CD31, S-100 protein, bcl-2, vimentin, EMA, desmin and muscle-specific actin).

These are old cases, with some dead patients. But the ethical committee approved it. Each patient entering into the hospital agrees to allow the scientific use of their data. The study followed the rules of the ethical committee of our institution.

## Results

This study group consisted of 17 female and 10 male patients (F/M: 1, 7) with a mean age of 50,3 years (range: 19–82 years). All of the patients referred to our institution for management of palpable soft tissue masses of various sizes. Focal swelling was present in 26 cases (96,2%) while pain occurred in 15 cases (55,5%) and the mean duration of symptoms was 13,8 months (range: 2–48 months). Laboratory tests were unremarkable. Neither history of trauma nor infection was noted. The mean maximum diameter of the lesions was 8,7 cm (range: 3–18,8 cm). Twenty lesions (74%) were located at the lower extremities with the thigh being the most frequent site (14/20) followed by the buttock (4/20), the calf (1/20) and the foot (1/20). Five lesions (18,5%) were affecting the upper extremities, with the shoulder being the most common location (4/5) followed by the forearm (1/5). Also, two lesions (7,5%) involved the paravertebral area. All of the masses presented with round to oval shapes and well-defined margins. Twenty–five lesions (92,6%) were deep-seated (below and/or attached to inner surface of the fascia lata or the antebrachial fascia) and two (7,4%) were superficial masses (attached to the skin or in the subcutaneous area). Adjacent anatomic structures were mildly to markedly displaced by the aforementioned lesions.

**Figure 1:**
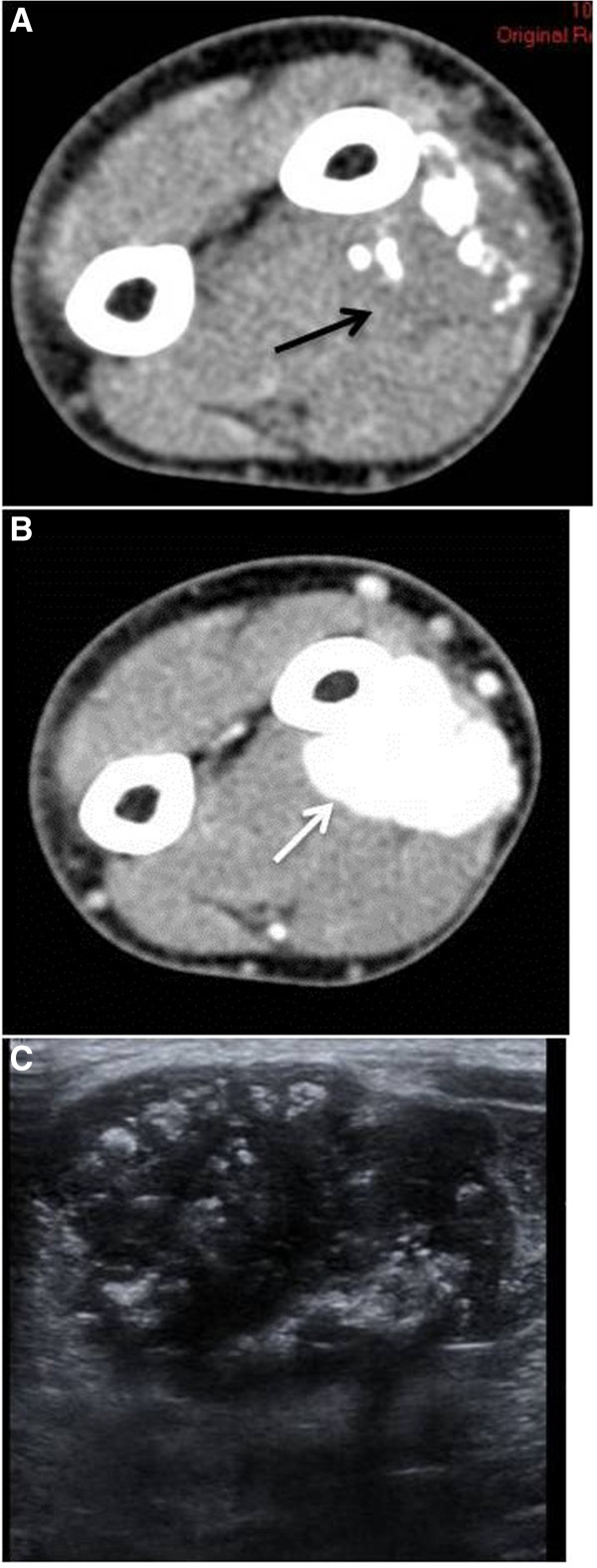
**Unenhanced CT scan (A) displays a deep soft tissue mass with internal calcifications lying adjacent to the diaphysis of the left radius. **The mass exhibits slightly lower attenuation compared to the neighboring muscles (black arrow). On contrast CT scan (**B**), the mass is homogeneously enhanced (white arrow). Ultrasound (**C**) demonstrates a hypoechoic mass containing multiple calcifications.

**Figure 2:**
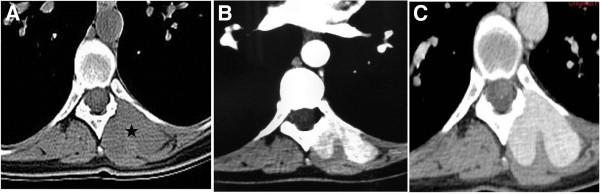
**Unenhanced CT (A) demonstrates a deep soft tissue mass in the left paravertebral area (asterisk). **The mass is isodense compared to muscle. No bone erosion is observed. At 30 s post-contrast infusion (**B**) the lesion exhibits mild heterogeneous enhancement while at 90 s post-contrast infusion (**C**) the lesion shows more intense homogeneous and prolonged enhancement.

**Figure 3:**
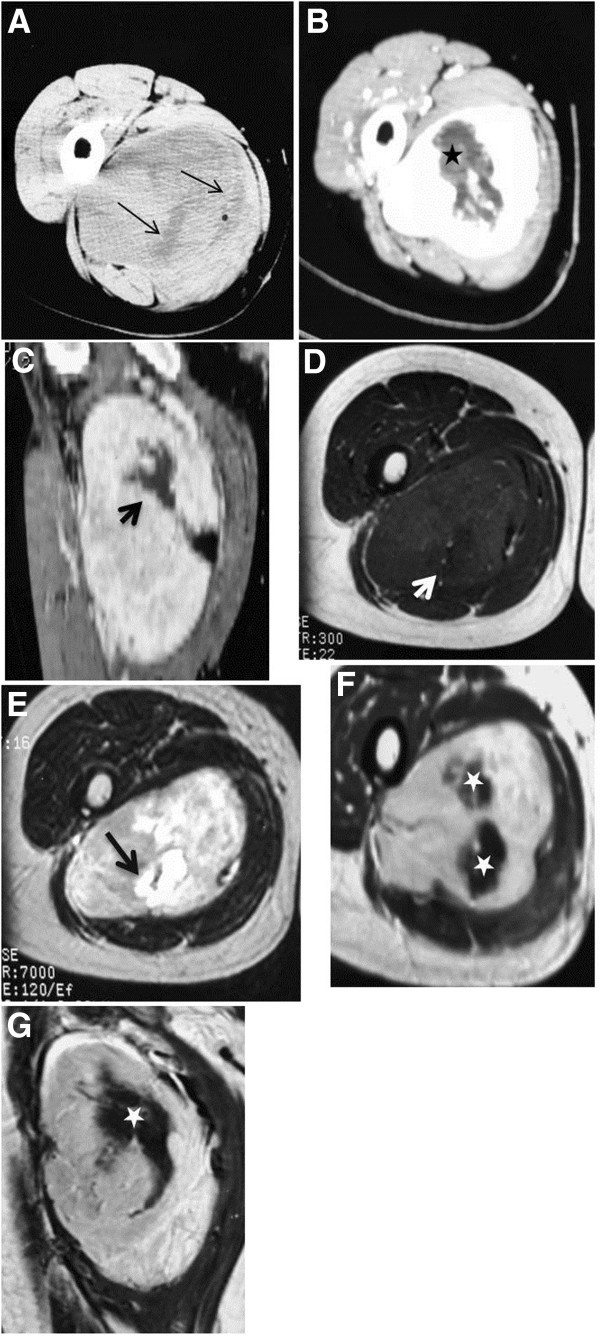
**Unenhanced CT scan (A) demonstrates a well**-**defined heterogeneous deep soft tissue mass containing scattered hypodense areas (thin arrows).** Enhanced CTimages (**B**–**C**) display heterogeneous enhancement. MR T1W image (**D**) displays an isointense mass relative to adjacent muscle containing an area of relative lower intensity (white arrow) that has markedly high SI on T2W MR image (**E**) (black thick arrow). Heterogeneous enhancement is depicted on enhanced T1MR images (**F**–**G**). Central unenhanced areas (asterisks) on both CT and MRI exams were of low SI on MRT1W images and high SI on T2W MR image.

**Figure 4:**
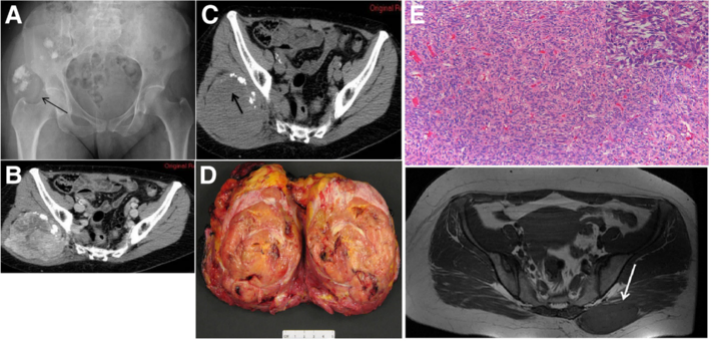
**(A) Radiograph demonstrates an ill defined and slightly radiodense area with multiple scattered calcifications of the right buttock (black arrow).** (**B**) Unenhanced CT scan displays a deep soft tissue mass with internal calcifications. (**C**) The mass is nearly isodense relative to adjacent muscle and contains (mainly in its upper portion (black arrow)) an ill-defined hypodense area that remained nearly unenhanced on post-contrast CT images. Gross specimen (**D**): The lesion is roundish with polycyclic margins, greyish to yellow in colour, and firm in consistency. Histology (**E**): Proliferation of bland-appearing spindle cells in a patternless-pattern either in a storiform or haphazard pattern set in a collagen stroma (Hematoxiline & Eosine, 10x magnification). In some areas the cells are arranged around stag-horn haemangiopericytoma-like vessels (Inset, Hematoxiline & Eosine, 20x magnification).

Of the eighteen lesions imaged with CT scan, five were hypodense (Figure 1) and two were isodense (Figure 2A) to adjacent musculature while eleven lesions appeared heterogeneous-mixed of iso and hypodense areas, on 1pre-contrast exams (Figures 3A, 4B). Attenuation values of the lesions were also higher than subcutaneous fat. Heterogeneous enhancement was evident in thirteen lesions while homogeneous contrast uptake was observed in four lesions (Figures 1B, 2C) (one case had no contrast administration). Septations were exhibited only in one CT case. All of the heterogeneously enhanced tumors contained markedly hyperdense portions admixed with patchy hypodense areas with mild to no enhancement (Figures 3B, 3C, 4C). In the dynamically –enhanced CT case, mild heterogeneous contrast uptake was depicted at 30 s and more intense prolonged enhancement was shown at 90 s and 180 s respectively (Figures 2B, 2C).

**Figure 5:**
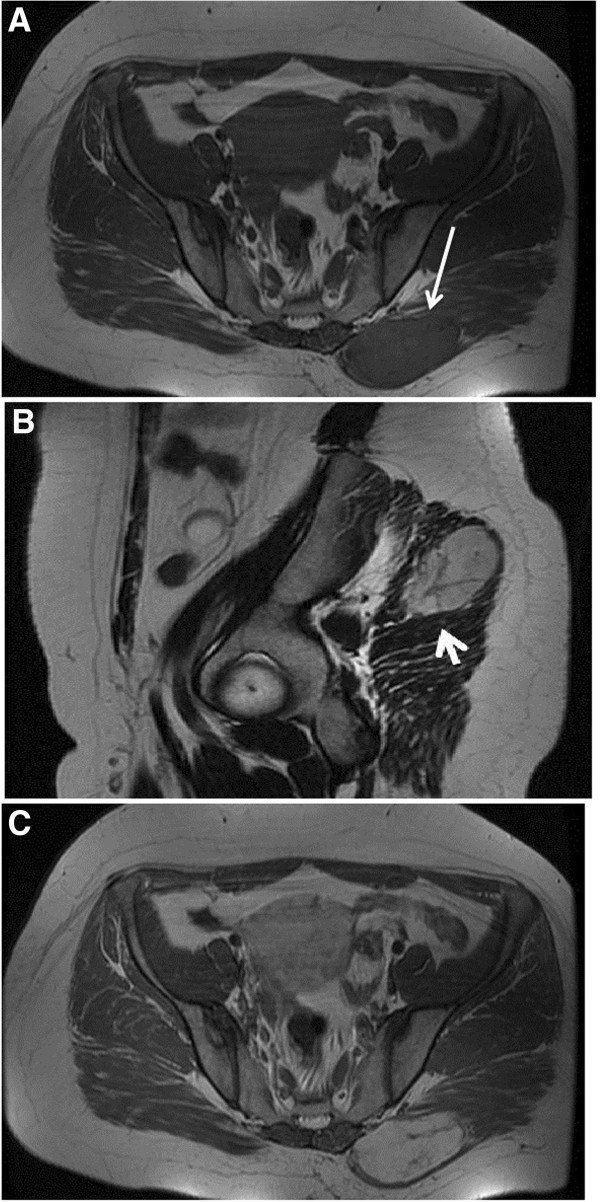
**A deep soft tissue mass is intramuscularly located at the right gluteus maximus muscle (white arrow). **The mass is isointense relative to adjacent muscle on T1 MR images (**A**). The mass has high SI and contains septations on T2MR image (**B**: white arrowhead). On gadolinium –enhanced T1 images (**C**), the lesion displays marked homogeneous enhancement. Internal septations remain unenhanced.

A total of fourteen cases were examined with MR imaging. On T1WSE, seven lesions were isointense to surrounding muscles (Figure 5A), one was hyperintense and five lesions were heterogeneous-mixed of iso and hypointense areas (Figure 3D). All lesions exhibited lower T1W signal intensity than subcutaneous fat. Of the nine tumors examined with T2WSE, seven lesions demonstrated mixed intensities with hyper and hypointense areas (Figure 3E) and two lesions were hyperintense (Figure 5B). Nine lesions failed to suppress and presented with heterogeneous/mixed intensities on T2 FSE fat sat and STIR images. Post-gadolinium exams revealed homogeneous and heterogeneous enhancement in four (Figure 5C) and six cases (Figures 3F, 3G) respectively. Patchy unenhanced areas were evident in six out of ten enhanced cases while irregularly shaped areas of low to markedly high signal intensities were additionally observed on T2WSE images of the four cases that were not imaged with gadolinium. Septations were noticed in eight MRI exams (Figures 5B, 5C). In total, patchy areas with mild or no enhancement on CT and T1WSE images and areas of low to markedly high signal intensities on T2WSE were observed in 19 (70,3%) out of 27 cases.

Ultrasound examination of five cases displayed three hypoechoic (Figure 1C) and two heterogeneous lesions of admixed hypo and slightly hyperechoic areas.

Six available radiographs showed ill defined mildly radio-dense areas in the soft tissues with or without scattered internal calcifications (Figure 4A). Gross calcifications were exhibited in six (22,2%) lesions. Additional file 1: Table S1–S3 display the imaging findings of the presented cases.

Grossly, the resected masses were round or oval in shape with well-defined margins. Cut surfaces were grayish-white to yellow (Figure 4D). Microscopically, tumors were made of haphazardly arranged spindle cells admixed with collagenous tissue of variable vascularity (Figure 4E). The diffused network of capillaries, totally collapsed or wide open sinusoids, are surrounded by compact proliferation of cells with oval nuclei, distinct nuclear membrane, granular chromatin, small nucleolus, ill-defined cytoplasm, thick reticular fibers all around. The cells were organized in a co-called “patternless” pattern. There is no strict correlation between morphology and behaviour. However, histological aspects that could predict an aggressive and malignant behaviour are: high cellularity, cellular atypia, extensive necrosis, >4 mitotic figures/10 HPF. Considering these aspects, aggressive features were present in 55,5% (15/27) of cases with necrosis in 22,2% (6/27) and degenerative changes were shown in 7,4% (2/27) of cases. On Immunohistochemistry, positive reaction was documented in 91,3% of cases for CD34, 60% of cases for CD99, 95,4% of cases for bcl-2 and 87,5% of cases for vimentin.

## Discussion

SFTs are rare neoplasms that usually involve the pleura or other serosal surfaces. Its origin has been controversial. Previous experimental reports showed that submesothelial cells had the ability to differentiate into mesothelial cells [34,35]. Despite they are frequently regarded as pleural tumors, a variety of extrathoracic locations has been reported like the meninges and the soft tissues of the neck [8-10], pelvis [11,16,17] and retroperitoneum [14,15], liver [12,13], and the soft tissue [18-33]. Soft tissue SFTs still represent a rare entity with 46 cases described in the English literature, mainly from a histopathological point of view. They principally affect adults between the fourth and seventh decades of life [16,17]. Clinically, extrathoracic SFTs cause symptoms that relate to tumor’s size and location. Systemic symptoms such as hypoglycemia, arthralgia, osteoarthritis, and clubbing have also been reported. These symptoms usually subside upon tumor’s resection [17]. In our cases, apart from focal swelling and mild to marked displacement of neighboring anatomic structures (e.g. muscles, neurovascular bundles), no other regional or systemic symptoms occurred and laboratory analysis ranged within normal limits.

Radiological findings concerned twenty seven well-defined masses with round or ovoid shapes displacing adjacent anatomic structures. On unenhanced CT images, pleural SFTs appeared isodense relative to muscle and some contained hypodense areas, while on enhanced CT images they usually exhibited mild to marked heterogeneous contrast uptake [35,36]. According to the Rosado-de-Christenson et al. study [36], isodensity on pre contrast CT scans correlates with hypercellular areas and capillary networks while markedly enhanced regions represent hypervascular areas, intermediate enhanced areas correlate with hypocellularity, and patchy hypodensities correspond to necrotic, myxoid or cystic changes. Of our eighteen lesions imaged with CT scans, two were isodense, five appeared relatively hypodense compared to neighboring muscles and eleven were mixed of isodense and hypodense areas. On enhanced images thirteen lesions were markedly and heterogeneously enhanced including patchy unenhanced areas (Figures 3B, 3C, 4C) while only four exhibited a more homogeneous enhancement pattern (Figures 1B, 2C). In reference to the dynamic characteristics, Fuksbrumer et al. [12] and Moser et al. [13] stressed the importance of marked and delayed enhancement and washout in characterizing liver SFTs. In our study, one case (Figure 1B) that was assessed with dynamic CT protocols, presented with mild arterial enhancement and more intense and prolonged enhancement on late venous and delayed phases. These features related histologically to cellular areas (prolonged enhancement) admixed with collagenous stroma (delayed enhancement).

According to the published MR features of SFTs, isointensity to muscle is most commonly displayed on T1-weighted sequences along with an increase of signal intensity on post gadolinium images and variable appearance on T2-weighted sequences. It is considered that the variety of signal intensity on T2-weighted images depends upon the different nature of the tumor components, namely, the amount of collagen and cellularity (fibroblasts), and on the presence of degeneration [9,34]. This series included seven masses of equal signal intensity on T1-weighted images relative to muscle (Figure 5A) and five lesions of heterogeneous-mixed intensity (Figure 3B, 3C). In most MR studies on mature fibrous tissue, the observed hypointensity on both T1 and T2-weighted sequences is related to the presence of hypocellularity and abundant collagenous stroma whereas fibrous tissue of relative increased cellularity and vascularity increases the signal intensity on T2-weighted sequences (Figure 5B) [34,37,38]. The aforementioned was noticed in all nine lesions that showed mixed to high signal intensities on T2-weighted images. Gadolinium enhancement of SFTs is generally a result of tumor vascularization that may vary from hypovascularity to hypervascularity. Of the ten cases that received gadolinium, six revealed intense heterogeneous enhancement and the remaining four cases were more homogeneously enhanced (Figure 5C). Similarly to the Tateishi et al. study [34], the enhancing portions corresponded histologically to areas of abundant cellularity combined with microvessels. Patchy heterogeneous areas of variable size were depicted in totally ten MRI exams (Figures 3F, 3G). Four of these cases demonstrated areas of low to intermediate and markedly high signal intensity on T2WSE and the remaining six showed areas of mild to no uptake on post contrast images and were correlated to hypocellular collagenous stroma (n=10) with co-existing necrotic (n=3) or degenerative (n=2) changes. Hypocellular collagenous stroma was also correlated with the patchy unenhanced areas of the remaining nine (n=9) contrast CT cases with co-existing necrotic changes in only one case (n=1). On histologic analysis there were additionally two other cases with necrotic changes; one of which was imaged solely with MRI and appeared homogeneous both on pre and post gadolinium images and the other was imaged only with U/S and contained a few scattered hypoechoic areas. Moreover, thin hypointense on T2WSE, linear or curvilinear structures were observed in eight out of fourteen MRI cases. These structures remained unenhanced and were attributed to smooth hypocellular fibrous septations. Calcifications are suggestive of necrosis but they are only sporadically reported in extrathoracic SFTs [17]. Likewise in this study, it was depicted in only six cases (22,2%).

The sonographic appearance of extremity SFTs is not pathognomonic and in this series, generally comprised of a heterogeneous hypoechoic, well defined mass, with or without calcifications deeply located in the soft tissues. Similarly, X-rays findings were not specific and mainly include that of a radio-dense soft tissue mass with ill defined margins and occasionally gross calcifications. In one lesion, DSA revealed marked vascularity of the lesion originating from the superficial and deep femoral vessels.

Histologically SFTs are well circumscribed and consist of a varying number of spindle cells randomly arranged in a collagenous background of variable vascularity. Some cases present with a hemangiopericytoma-like pattern of irregular branching vessels and occasionally storiform or herringbone patterns (Figure 4E) are seen [9]. Immunohistochemistry is essential in differentiating these tumors from other spindle-cells neoplasms [16]. Positivity for CD34, CD99 and bcl-2 is an indicator of SFTs [3,16]. Pathological criteria for aggressiveness include tumor size >5 cm, high cellularity, nuclear pleomorphism, high mitotic rate (more than 4 mitosis in 10 HPF) and necrosis [32]. In the present study 55,5% of cases (15/27) contained aggressive features; one tumor (max diameter: 5,2 cm) locally recurred at 66 mo postoperatively, one case (max diameter: 15 cm) presented with concomitant lung metastases and two others (max diameters: 7 cm and 8,6 cm) metastasized postoperatively (to bones, lungs and soft tissues) at 46 mo and 85 mo respectively. The first lesion that locally recurred at 66 mo follow-up was characterized by a homogeneous enhancing pattern on CT and MRI images and showed no evidence of aggressiveness while the lesion with concomitant metastases showed aggressive features on histology and hypodense-unenhanced areas on post contrast CT. The remaining two cases that metastasized postoperatively were characterized as aggressive on histological grounds while one of them was homogeneously enhanced and the other was heterogeneously enhanced on CT scans. As a result, we can not predict the biological behavior of soft tissue SFTs based solely either on histopathologic or imaging evaluation.

The behavior of extrathoracic SFTs is unpredictable. Some histologically aggressive tumors behave in a benign fashion while some morphologically histologically benign lesions behave aggressively [16,32]. Ten to 13% of cases recur and/or metastasize [22,24]. They may also recur or metastasize after complete surgical resection even in the absence of atypical histological features. Therefore, complete surgical resection and long-term follow-up is recommended for patients with extrathoracic SFTs [16]. Moreover, Cranshaw et al. [33] showed that extrathoracic SFTs have a higher rate of malignant behavior than that classically described and recommended that in the presence of atypical features extrathoracic SFTs should be managed and followed up in the same manner as other high-grade soft tissue tumors, which is not what we found in our cases.

In conclusion, imaging features of extremity SFTs lack specificity and correlate with the variable histopathological appearances. However, soft tissue SFTs often (16/27, 59,2%) are isodense and isointense masses mixed with hypodense and hypointense areas on unenhanced CT and MR T1W. Depending upon tumor’s cellularity, vascularity, collagen distribution and/or degeneration, heterogeneous enhancement and areas of mixed to high signal intensity on MRT2W are observed. Although rare, a high index of suspicion of a soft tissue SFT should be raised when a deep heterogeneous-mixed soft tissue mass with marked enhancement is encountered.

## Competing interests

The authors declare that they have no competing interests.

## Authors’ contributions

ZP made and wrote the article. MA and MG checked the histology. PP checked the global scientific work. ES checked the surgical part. FG drafted the manuscript. DV proposed the subject and checked the work. All authors read and approved the final manuscript.

## Supplementary Material

Additional file 1: Table S1 CT findings. **Table S2. **MR findings. **Table S3. **US findings. Click here for file
