# Supplementary material for: Solitary fibrous tumors of the soft tissues: imaging features with histopathologic correlations
Source: Clin Sarcoma Res. 2013 Jan 25;3:1. doi: 10.1186/2045-3329-3-1 (PMC3637805; doi:10.1186/2045-3329-3-1)
Supplement: Additional file 1: Table S1 — CT findings. Table S2. MR findings. Table S3. US findings. [file 2045-3329-3-1-S1.docx]

***Table S1. CT findings.***

| **Computed Tomography** | **Attenuation** | **Cases no :18** |
| --- | --- | --- |
| Pre-Contrast* | Low  Iso  Mixed | 5  2  11 |
| Post-Contrast | Homogeneous  Heterogeneous | 4  13 |
| **Internal Morphology** | | |
| Septation  Patchy Areas  Calcifications |  | 1  13(9 only on CT and 4 on CT+MRI)  6 |

***Table S2 : MR findings.***

| **MRI Sequence** | **Intensity** | **Cases no: 14** |
| --- | --- | --- |
| T1-Weighted* | Iso  High  Mixed | 7  1  5 |
| T2-Weighted | High  Mixed | 2  7 |
| T2 FAT SAT/STIR | High/Mixed | 9 |
| Enhancement | Homogeneous  Heterogeneous | 4  6 |
| **Internal Morphology** | | |
| Septation  Patchy Areas |  | 8  **10** *(6 on Gd MRI+4 on T2W)* |

***TableS 3 : US findings.***

| **Ultrasound** | **Cases no :5** |
| --- | --- |
| Hypoechoic  Hyperechoic  Mixed  Calcifications | 3  -  2  2 |
